# On a New Variety of the Dislocation of the Humerus

**Published:** 1846-11

**Authors:** 

**Affiliations:** Tubingen


*On a new variety of the dislocation of the Humerus.*—By Professor Roser, of Tubingen.—The professor discovered in a subject intended for dissection at the University of Tubingen, a variety of dislocation of the humerus not hitherto described by any author. The head of the humerus lay in front of the short head of the biceps ; the sub-scapular muscle was ruptured and completely detached from the lesser tuberosity of the humerus, and the head of the bone elevated the scapular extremity of the pectoralis minor. In ordinary dislocations of the shoulder, the upper extremity of the humerus liesimmediately on the outer border of the scapula ; in this dislocation it separated from it by the biceps and the coraco-brachialis, whose tendons pass behind instead of in front of the humerus. On a careful examination the following dispositions were observed.

The dislocated limb was abducted and slightly everted, the head of the humerus touching the inferior border of the coracoid process. Intimate adhesions existed between the muscles of the shoulder and the ligamentous apparatus of the joint. The brachial plexus was surrounded by a very dense cellular tissue. The sub-scapular muscle, detached from the lesser tuberosity of the humerus, terminated in a bulbous mass which rested on the neck of the scapula, and involved the musculo-cutaneous nerve. The head of the humerus lay in a capsule of new formation below and internal to the coracoid process, was tolerably moveable, and the pectoralis minor was expanded over it, adhering very firmly to its capsule. The tendons of the coraco-brachialis and short head of the biceps descend behind the head of the humerus involved in the capsule. The long head of the biceps was entirely displaced from its groove, described a curve round the head of the humerus, and was completely adherent to and confounded at its insertion with the fibrous tissue which filled the glenoid process.

The subject of this observation had fallen seven years previously while carrying a heavy load up a hill. The first attempts at reduction were made by some wood-cutters who came to his assistance.—Several surgeons to whom he applied on the following day were equally unsuccessful. Very forcible tractions and several methods, including that of De la Mothe, were tried, but every time that the head of the bone was thought to be reduced it was found to be still displaced. The patient regained tolerable use of the limb, being able to dig and thrash, but could not put his coat on the left arm. He often experienced pain and a sense of numbness in the fingers.

Professor Roser easily produced this species of dislocation on the dead body, by cutting the tendon of the sub-scapular muscle, displacing the long tendon of the biceps from its groove, and then forcing the head of the humerus downwards. But what (he asks) was the obstacle to reduction? It was not, he thinks, muscular action, but the interposition of the short head of the biceps between the head of the humerus and the glenoid cavity. To reduce this dislocation, the same principle should be adopted that is followed in other reductions—viz., to cause the head of the bone in re-entering to follow the course it took in its exit, and as while being displaced it probably sustained violent torsion outwards, during reduction it should be forcibly rotated inwards, so as to slide over or else displace the interposed soft parts.

*Ibid, from Archiv. für Physiologie Heilk von Roser.*

